# Circular RNA circPIP5K1A contributes to cancer stemness of osteosarcoma by miR-515-5p/YAP axis

**DOI:** 10.1186/s12967-021-03124-6

**Published:** 2021-11-13

**Authors:** Pengxu Shi, Yueting Li, Qingsheng Guo

**Affiliations:** 1 Department of Bone Surgery, The People’s Hospital of Liaoning Province, No. 33 Wenyi Road, Shenhe District, Shenyang, 110016 Liaoning People’s Republic of China; 2 Department of Breast Surgery, Cancer Hospital of China Medical University, No. 44 Xiaoheyan Road, Dadong District, Shenyang, 110042 Liaoning People’s Republic of China

**Keywords:** Osteosarcoma, Stemness, CSCs, circPIP5K1A, miR-515-5p, YAP

## Abstract

**Background:**

Osteosarcoma is a common type of bone tumors and frequently occurs in children and adolescents. Cancer stem cells (CSCs) are a unique sub-type of self-renewal cancer cells and the stemness of cancer cells are involved in the spread, recurrence, metastasis, and even therapeutic resistance. However, the regulation mechanisms of CSCs in osteosarcoma are poorly understood. Circular RNA (circRNA) is a unique sort of non-coding RNAs and widely participate in the modulation of cancer progression.

**Methods:**

In this study, we identified the critical function of circular RNA circPIP5K1A in stemness of osteosarcoma cells.

**Results:**

CircPIP5K1A expression was significantly enhanced in clinical osteosarcoma tissues compared with the adjacent normal tissues. The depletion of circPIP5K1A by siRNA repressed osteosarcoma cell viabilities and induced osteosarcoma cell apoptosis. The suppression of circPIP5K1A attenuated the capabilities of invasion and migration of osteosarcoma cells. The circPIP5K1A knockdown increased E-Cadherin expression and decreased Vimentin expression in osteosarcoma cells. The sphere formation abilities of osteosarcoma cells were repressed by the depletion of circPIP5K1A. The CD133^+^CD44^+^ cell population of osteosarcoma cells was reduced by circPIP5K1A knockdown. The expression of ALDH1 and Nanog was decreased by the inhibition of circPIP5K1A in osteosarcoma cells. Mechanically, circPIP5K1A enhanced YAP expression by targeting miR-515-5p. MiR-515-5p inhibited stemness of osteosarcoma cells. The CSCs properties of osteosarcoma cells were repressed by circPIP5K1A knockdown or miR-515-5p mimic, while miR-515-5p inhibitor or YAP overexpression reversed circPIP5K1A knockdown-induced repression. Tumor xenograft analysis in nude mice demonstrated that the depletion of circPIP5K1A represses osteosarcoma cell growth in vivo.

**Conclusion:**

In conclusion, we identified that circular RNA circPIP5K1A contributed to cancer stemness of osteosarcoma by miR-515-5p/YAP axis. Targeting circPIP5K1A may be considered as a potential therapeutic strategy for osteosarcoma treatment.

## Background

Osteosarcoma is a form of bone tumor most frequently occurs in children and adolescents, especially in children [1]. The standard treatments for osteosarcoma include surgical operation with or without radiation therapy, as well as chemical therapy [2]. Despite the favorable outcomes for patients with localized tumor, patients with advanced or metastatic osteosarcoma present poor prognosis [2]. The acquired resistance to standard therapy highlights the urgent need to discover novel therapeutic regimens. Cancer stem cells (CSC) is a unique sub-type of self-renewal cancer cells that participate in the spread, recurrence, metastasis, and even therapeutic resistance [3]. In some cases, patients with osteosarcoma who has finished the first therapy would somehow develop a distant metastasis, which implies the possible involvement of CSCs [4]. Targeting the stemness of osteosarcoma maybe a promising area to discover new therapeutical targets.

Circular RNA (circRNA) is a unique type of RNAs presented as a covalent closed loop other than the regular linear structure [5]. CircRNAs were initially thought to be low expressed in cells and an error during RNA splicing [6]. Nevertheless, the development of high throughput sequencing technologies have led to identification of a certain number of circRNAs differently expressed in cancer cells [6]. It is now widely accepted that circRNAs usually act as sponges for microRNAs (miRNAs) to participate in various cellular behaviors, including cell proliferation, differentiation, apoptosis and migration, etc., and are even recognized as oncogenes or tumour suppressors [6]. Among the functional circular RNAs, circPIP5K1A is proposed to facilitate the progression of several cancers, including gastric cancer [7], ovarian cancer [8], non-small cell lung cancer (NSCLC) [9], colon cancer [10], and glioma [11]. However, the function of circPIP5K1A in osteosarcoma is not yet identified.

MiRNAs are short-length, noncoding RNAs that interact with the 3′-untranslated region (3′-UTR) regions of mRNAs, which suppress the stability or translation of target mRNAs [12]. MiRNAs have been proposed to participate in the carcinogenesis and progression of various cancers, acting as oncogenes or suppressors depending on their target mRNAs [13]. MiR-515-5p is indicated as a tumor suppressor in several cancers. For example, low expression of miR-515-5p expression is correlated with advanced tumor stage of prostate cancer patients [14]. Pardo and colleagues also suggested that miR-515-5p overexpression inhibited tumour cell metastasis through targeting MARK4 in breast cancer and non-small cell lung cancer model [15]. Nevertheless, the precise role of miR-515-5p in osteosarcoma and the involved mechanisms remains to be elucidated.

Yes-associated protein1 (YAP1) is a critical effector for signaling transduction of Hippo pathway, which participates in the proliferation, differentiation, migration, invasion, and self-renewal of cells [16]. It has been testified that YAP1 is frequently amplified in multiple cancers and could promote chemoresistance through inactivating the oncogenic drivers [17, 18]. RNA sequencing and survival analysis suggested that YAP/TEAD is a potential therapeutic target for osteosarcoma [19]. In this study, we proposed that cirPIP5K1A could affect stemness of osteosarcoma through interacting with miR-515-5p and enhance expression of YAP1. Targeting cirPIP5K1A may become a promising therapy for cure of osteosarcoma.

## Materials and methods

### Patient samples

This work was approved by the Ethics Committee of the People’s Hospital of Liaoning Province. Forty-five paired osteosarcoma tumor tissues and adjacent normal tissues were collected from osteosarcoma patient. All participants have signed the written informed consent.

### Cell culture and transfection

Human osteosarcoma cell line MG63, 143B, U2OS and Saos2 were purchased from Shanghai Institutes for Biological Sciences Cell Resource Center (China). The cell lines in this study were validated. The MG63 and 143B cell line were cultured in MEM medium (Gibco, USA) containing 10% FBS (Hyclone, USA) plus 1% penicillin and streptomycin (Solarbio, China). The U2OS and Saos2 cells were maintained in McCoy’s 5A medium (Hyclone) added with 10% FBS (Hyclone) plus 1% penicillin and streptomycin (Solarbio). All cells were placed in a 37 °C incubator with 5% CO_2_. The siRNA targeting circPIP5K1A (si-PIP5K1A), miR-515-5p mimics and inhibitor and the negative controls (NC), andYAP1 overexpression plasmid (pCMV-YAP1) were purchased from RiboBio (China). The MG63 and U2OS cell were seeded in 6-well plates, and cultured overnight to form a 60% monolayer, and transfected by the pCMV-YAP1 or RNA sequences through a lipofectamine 2000 (Invitrogen, USA) following manufactures’ description.

### Cell viability

Cell viability of MG63 and 143B was detected by a MTT experiment. In brief, MG63 and 143B cells were transfected with siPIP5K1A or the NC, and seeded into a 96-well plate (5000 cells/well). Next, 10 µL MTT solution (Sigma, 5 mg/mL) was added into each well at indicated time, and incubated for 4 h. After that, the medium was replaced by 150 µL DMSO and the plates were shaken for 10 min in dark. A microplate detector (PerkinElmer, Germany) was used to detect the absorbance values at 490 nm.

### Flow cytometry

Flow cytometry was performed to determine cell apoptosis and CD133^+^CD44^+^ cell population. MG63 and 143B cells were transfected with siPIP5K1A or the NC for 48 h, and were collected for further staining. To detect apoptotic cells, the collected cells were stained by an Annexin V-FITC/PI detection kit (Beyotime, China) following manufacturer’s instruction. In Brief, the cells were stained by Annexin V and PI at room temperature in dark for 15 min, subsequently washed with PBS and detected in flow cytometer (BD Biosciences, USA). To determine the portion of stem-like cells, MG63 and 143B cells were incubated with FITC conjugated anti-CD133 (Thermo, USA) antibody and PE-conjugated anti-CD44 antibody (Thermo) and the positive staining was detected by flow cytometer.

### Transwell assay

Transwell chambers (Corning, USA) were used to measure cell migration and invasion. Briefly, MG63 and 143B cells transfected with siPIP5K1A or NC were suspended in medium containing no FBS, and were plated in the upper chamber. The lower chamber was filled with medium containing 10% FBS. The invaded cells were fixed in 4% PFA after 24-h incubation, stained with crystal violet (0.2%) for 20 min, photographed by a microscope (Leica, Germany) and counted.

### Sphere formation

MG63 and 143B cells (500 cells/well) were seeded into a low-attachment culturing 24-well plate (Corning, USA), in a medium mixture which contains DMEM/F12 (Hyclone, USA), methylcellulose (Sigma), B27 (Sigma), bFGF (20 ng/mL, Sigma), and EGF (10 ng/mL, Sigma). After a 10-day culturing in 37 °C incubator, the formed spheres were captured under light microscope (Leica) and counted. The quantification the spheres was analyzed using Image J software.

### RNA extraction and quantification

MG63 and 143B cells were subjected to indicated transfection and lysed by a TRIzol reagent (Beyotime) to extract total RNA. The RNAs were reverse-transcribed to cDNAs by using a cDNA synthesis kit (TransGen, China). Next, the quantification of genes was conducted with a SYBY Green Mixture kit (Thermo). U6 and β-actin were used as internal control for normalization of YAP1, circPIP5K1A, miR-515-5p, separately. The relative level of genes was calculated via a 2^−ΔΔCt^ method. The primers were listed below:

circ PIP5K1A: sense, 5′-AGATTCCCTAACCTCAACCAGA-3′, antisense, 5′-CGAATGTTCTTGCCACCTGC-3′;

miR-515-5p: sense, 5′-CGGGTTCTCCAAAAGAAAGCA-3′, antisense, 5′-CAGCCACAAAAGAGCACAAT-3′;

YAP1: sense, 5′-CAAGACCCATCGGACTGACAG-3′, antisense, 5′-AGCCATAAGCATCAGCTCATTTT-3′;

U6: sense, 5′-AGGGGCCATCCACAGTCTTC′, antisense, 5’-AACGCTTCACGAATTTGCGT-3′;

β-actin: sense, 5′-TGGGTGTGAACCACGAGAA-3′, anti-sense, 5′-GGCATGGACTGTGGTCATGA-3′;

E-Cadherin: sense, 5′-AAAGGCCCATTTCCTAAAAACCT-3′, antisense, 5′-TGCGTTCTCTATCCAGAGGCT-3′;

Vimentin: sense, 5′-GCTGCGAGAGAAATTGCAGGA-3′, antisense, 5′-CCACTTTCCGTTCAAGGTCAAG-3′;

ALDH1: sense, 5′-ACCTCTCACCGCCCTTTATCT-3′, antisense, 5′-GTGAAGGCGATCTTGTTGATCT-3′;

Nanog: sense, 5′-TTTGTGGGCCTGAAGAAAACT-3′, antisense, 5′-AGGGCTGTCCTGAATAAGCAG-3′.

### Western blotting

MG63 and 143B cells were subjected to indicated transfection and lysed by RIPA lysis solution (SolarBio) with cocktail of protease inhibitors (Sigma) to extract proteins. A total of 35 μg proteins was resolved in SDS-PAGE, shifted to PVDF membranes, and soaked in 5% nonfat milk for 1.5 h. The membranes were incubated with specific primary antibodies against E-Cadherin (1:1000, Abcam, USA), Vimentin (1:1000, Abcam), β-actin (1:1000, Abcam), ALDH1 (1:1000, Abcam), Nanog (1:1000, Abcam), separately, overnight at 4 °C. Subsequently, the membranes were incubated with HRP-conjugated anti-mouse or anti-rabbit (1:2000, Abcam) for 45 min. The visualization of bands was achieved by using an ECL solution (Millipore, USA).

### Luciferase reporter gene assay

The binding site prediction was performed by an online tool TargetScan (http://www.targetscan.org/vert_71/). The 3′UTR region of YAP1 and circPIP5K1A were synthesized and cloned into pmirGLO vector (Promega, USA) to obtain YAP1-WT and PIP5K1A-WT. Similarly, the mutated luciferase reporter vectors of circPIP5K1A (PIP5K1A-Mut) and 3′UTR of YAP1 (YAP1-Mut) were obtained by inserting corresponding sequences into pmirGLO vector. MG63 and 143B cells were co-transfected with the WT or Mut vectors with miR-515-5p mimics or NC for 24 h. Subsequently, the cells were lysed and the luciferase activity was detected by a dual-luciferase reporter system (Promega).

### Immunofluorescence analysis

Cells were solidified at 4% paraformaldehyde for 30 min, treated with Triton X 100 (0.2%) for 10 min and treated with BSA (2%) for 30 min. The slides were hatched with the primary antibody overnight at 4 °C, then hatched with secondary antibodies (Proteintech, China) for 1 h at 37 °C. The slides were stained with the DAPI (Beyotime, China) for 10 min at 25 °C. The Nikon microscope (Tokyo, Japan) was utilized to analyze the immunofluorescence.

### Tumor xenograft

BALB/c nude mice aged five-week were obtained from Charles River Laboratories (China). Osteosarcoma cells were transfected with siPIP5K1A or NC, and subcutaneously injected in the right flank (1 × 10^6^ cells in 100 μL saline). The tumor size was measured at indicated time and calculated by the following formula: 0.5 × length  ×  width^2^. The experiments were approved by the Animal Care and use Committee of the People’s Hospital of Liaoning Province.

### Statistics

Data in this work were shown as mean  ±  SD and processed by a SPSS software (Version 22,0). Student’s *t *test or one-way ANOVA method was used for comparison between two or more groups. The differences were considered as statistically significant with P values  < 0.05.

## Results

### The expression of circPIP5K1A is enhanced in clinical osteosarcoma tissues and contributes to osteosarcoma cell proliferation in vitro

**Fig. 1:**
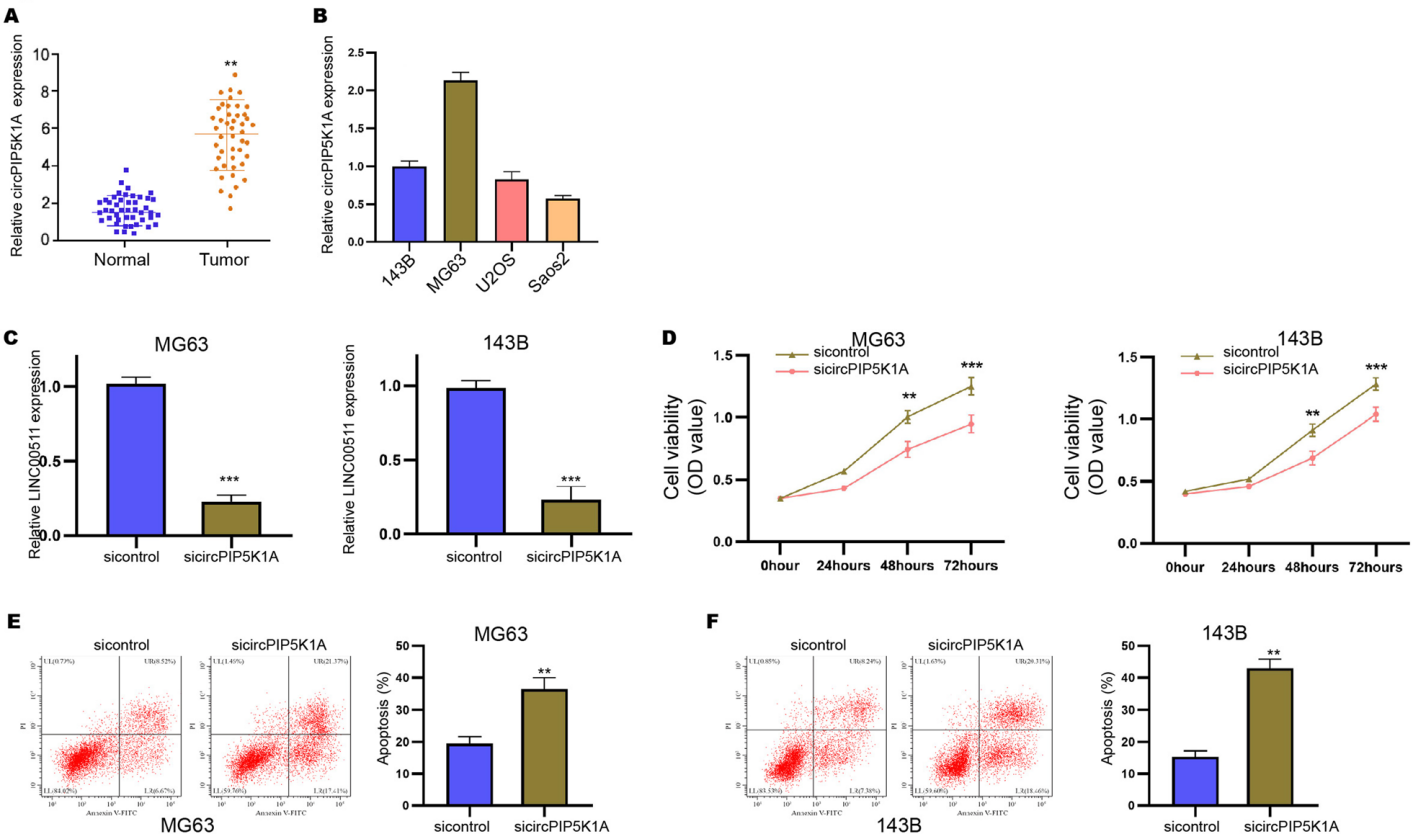
The expression of circPIP5K1A is enhanced in clinical osteosarcoma tissues and contributes to osteosarcoma cell proliferation in vitro. **A** CircPIP5K1A expression was determined by qPCR in clinical osteosarcoma tissues (n  = 45) and adjacent normal tissues (n  = 45). **B** CircPIP5K1A expression was assessed by qPCR in MG63, 143B, U2OS and Saos2 osteosarcoma cell lines. **C**–**F** MG63 and 143B cells were transfected with circPIP5K1A siRNA. **C** CircPIP5K1A expression was analyzed by qPCR. **D** Cell viability was examined by MTT analysis. **E**, **F** Cell apoptosis was tested by Annexin V-FITC/PI detection kit. Mean  ±  SD (**P  < 0.01)

In order to confirm the association of circPIP5K1A with osteosarcoma, we detected circPIP5K1A expression in clinical osteosarcoma samples. We found that circPIP5K1A expression was significantly enhanced in clinical osteosarcoma tissues (n  = 45) relative to the adjacent normal tissues (n  = 45) (Fig. 1A). Meanwhile, we assessed circPIP5K1A expression in osteosarcoma MG63, 143B, U2OS and Saos2 cell lines, in which MG63 and 143B cell lines presented the highest expression and were selected for the further experiments (Fig. 1B). The MG63 and 143B cells were transfected with circPIP5K1A siRNA and the effectiveness was verified (Fig. 1C). The inhibition of circPIP5K1A by siRNA repressed the MG63 and 143B cell viabilities (Fig. 1D). The apoptosis of MG63 and 143B cells was enhanced by the depletion of circPIP5K1A (Fig. 1E, F).

### The inhibition of circPIP5K1A represses invasion and migration of osteosarcoma cells

**Fig. 2:**
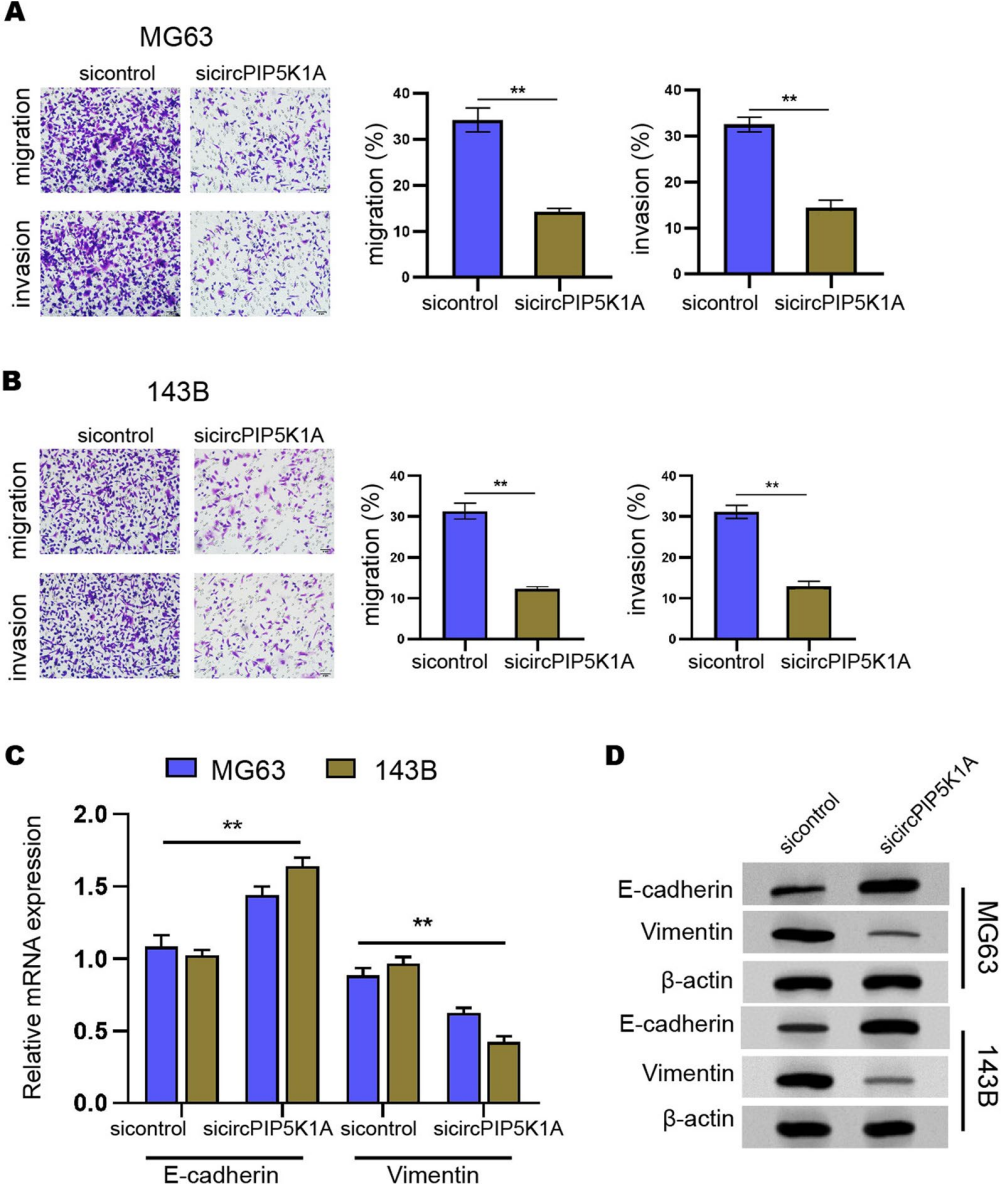
The inhibition of circPIP5K1A represses invasion and migration of osteosarcoma cells. **A**–**D** MG63 and 143B cells were transfected with circPIP5K1A siRNA. **A**, **B** Cell invasion and migration were analyzed by transwell assays. **C** The mRNA expression of E-Cadherin and Vimentin was detected by qPCR. **D** The protein levels of E-Cadherin and Vimentin were measured by Western blot. Mean  ±  SD (**P  < 0.01)

Furthermore, we observed that the suppression of circPIP5K1A by siRNA attenuated the capabilities of invasion and migration of MG63 and 143B cells (Fig. 2A, B). In addition, the mRNA expression of E-Cadherin was enhanced but Vimentin was reduced by circPIP5K1A knockdown (Fig. 2C). Consistently, the inhibition of circPIP5K1A increased E-Cadherin expression and decreased Vimentin expression at the protein levels in MG63 and 143B cells (Fig. 2D).

### The suppression of circPIP5K1A attenuates stemness of osteosarcoma cells

**Fig. 3:**
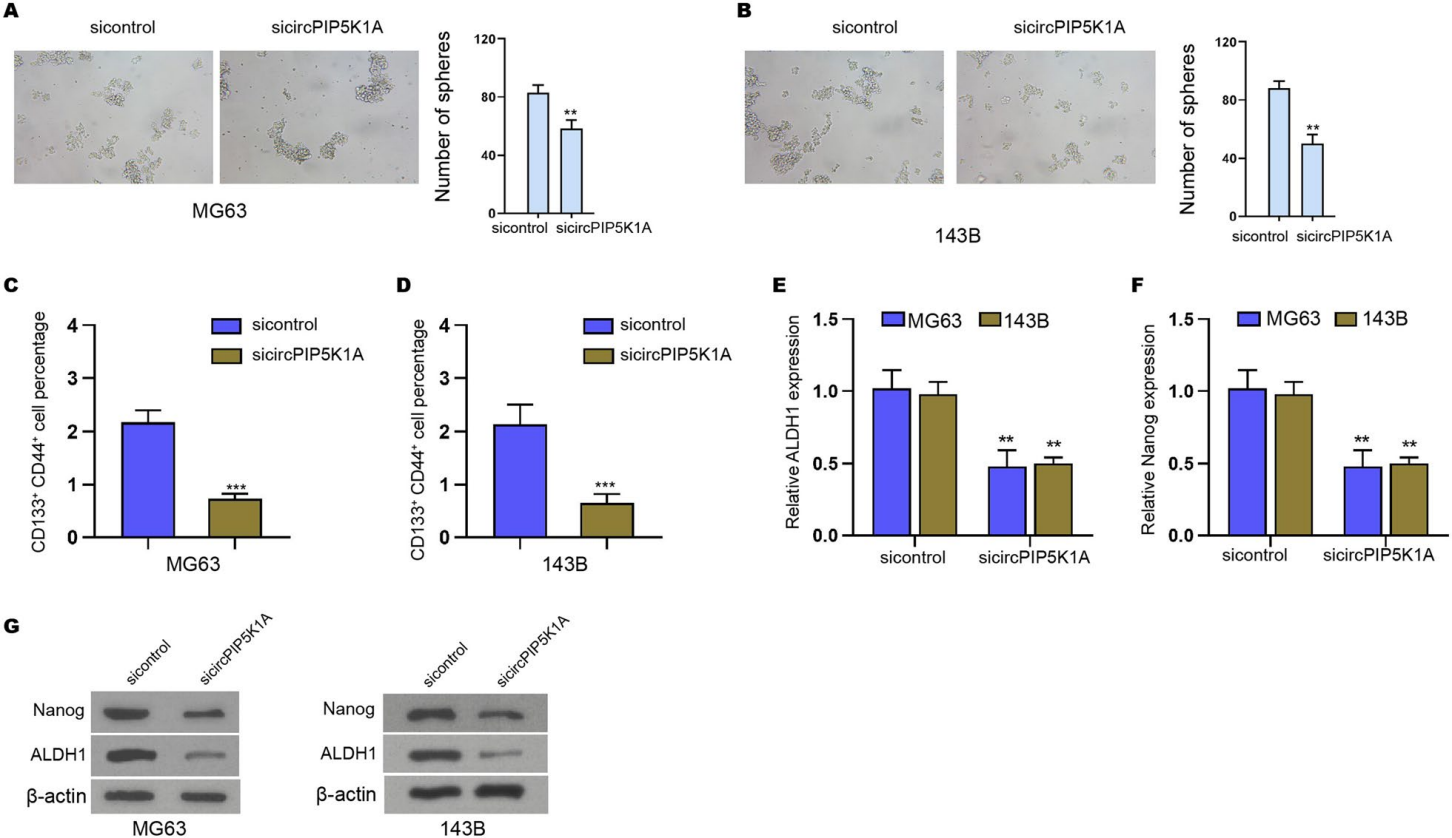
The suppression of circPIP5K1A attenuates stemness of osteosarcoma cells. **A**–**G** MG63 and 143B cells were transfected with circPIP5K1A siRNA. **A**, **B** The stemness was analyzed by sphere formation assays. **C**, **D** The CD133^+^CD44^+^ cell population was measured by flow cytometer. **E**, **F** The mRNA expression of ALDH1 and Nanog was determined by qPCR. **G** The protein levels of ALDH1 and Nanog were detected by Western blot. Mean  ±  SD (**P  < 0.01, ***P  < 0.001)

We then concerned about the effect of circPIP5K1A on the cancer stem cell properties of osteosarcoma cells. The sphere formation abilities of MG63 and 143B cells were repressed by the depletion of circPIP5K1A (Fig. 3A, B). Besides, the CD133^+^CD44^+^ cell population of MG63 and 143B cells was reduced by circPIP5K1A knockdown (Fig. 3C, D). Meanwhile, the mRNA expression of ALDH1 and Nanog was decreased by the inhibition of circPIP5K1A in MG63 and 143B cells (Fig. 3E, F). Similarly, the suppression of circPIP5K1A inhibited ALDH1 and Nanog protein levels in MG63 and 143B cells (Fig. 3G).

### MiR-515-5p is a target of circPIP5K1A in osteosarcoma cells

**Fig. 4:**
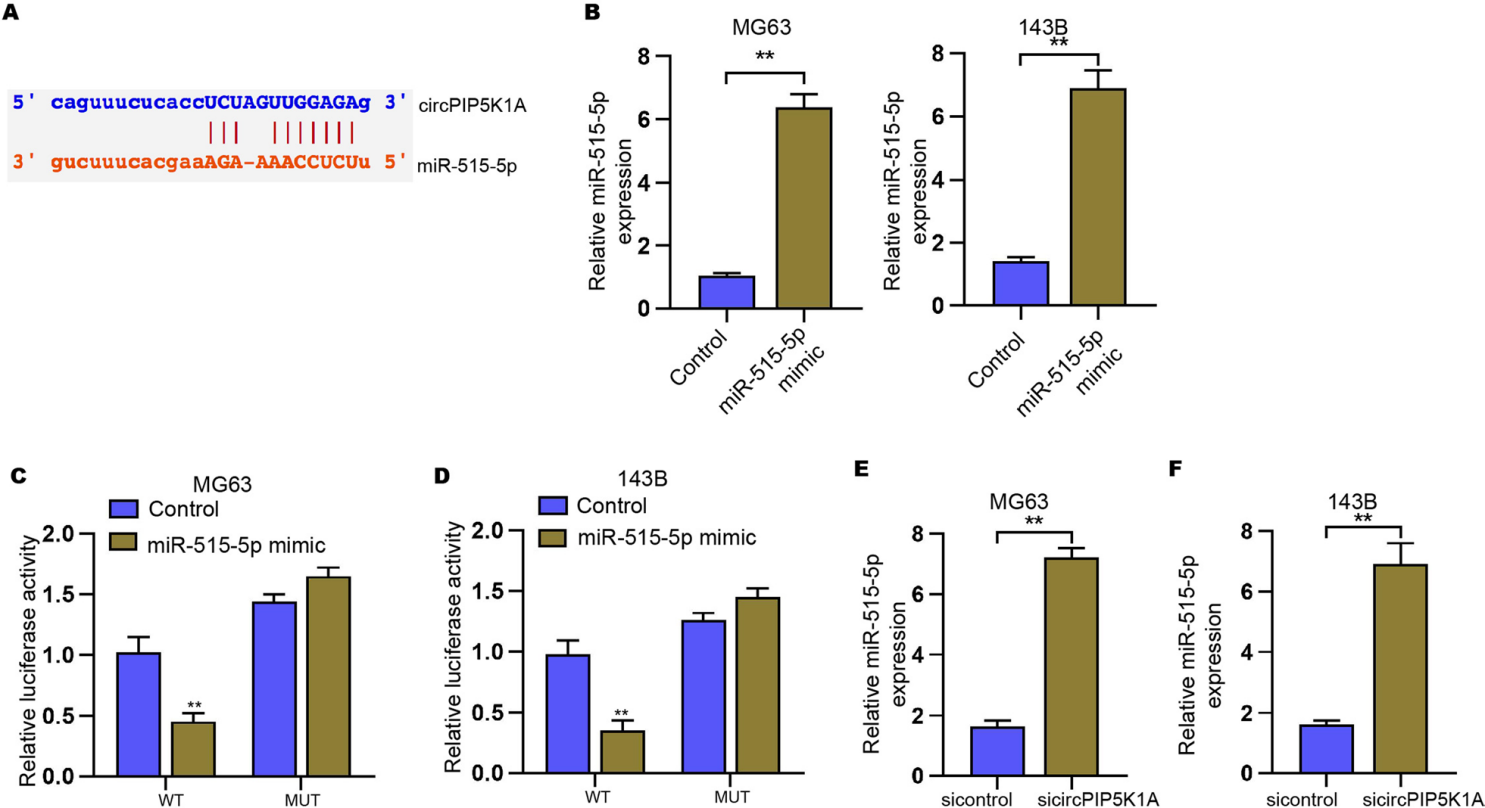
MiR-515-5p is a target of circPIP5K1A in osteosarcoma cells. **A** The interacted binding site between circPIP5K1A and miR-515-5p. **B**–**D** MG63 and 143B cells were transfected with miR-515-5p mimic. **B** MiR-515-5p expression was measured using qPCR. **C**, **D** Luciferase activities of circPIP5K1A or circPIP5K1A mutant (miR-515-5p binding site) were analyzed by luciferase reporter gene assay. **E**, **F** MiR-515-5p expression was determined using qPCR in MG63 and 143B cells transfected with circPIP5K1A siRNA. Mean  ±  SD (**P  < 0.01)

Next, we identified that miR-515-5p is one of the unreported targets of circPIP5K1A and found the binding site between miR-515-5p and circPIP5K1A (Fig. 4A). The expression of miR-515-5p significantly enhanced by the transfection of miR-515-5p mimic in MG63 and 143B cells (Fig. 4B). Meanwhile, we validated that the luciferase activities of circPIP5K1A, but not circPIP5K1A with miR-515-5p binding site mutant, were repressed by miR-515-5p mimic in MG63 and 143B cells (Fig. 4C, D). The depletion of circPIP5K1A by siRNA remarkably increased the miR-515-5p expression in MG63 and 143B cells (Fig. 4E, F).

### MiR-515-5p inhibits stemness of osteosarcoma cells

**Fig. 5:**
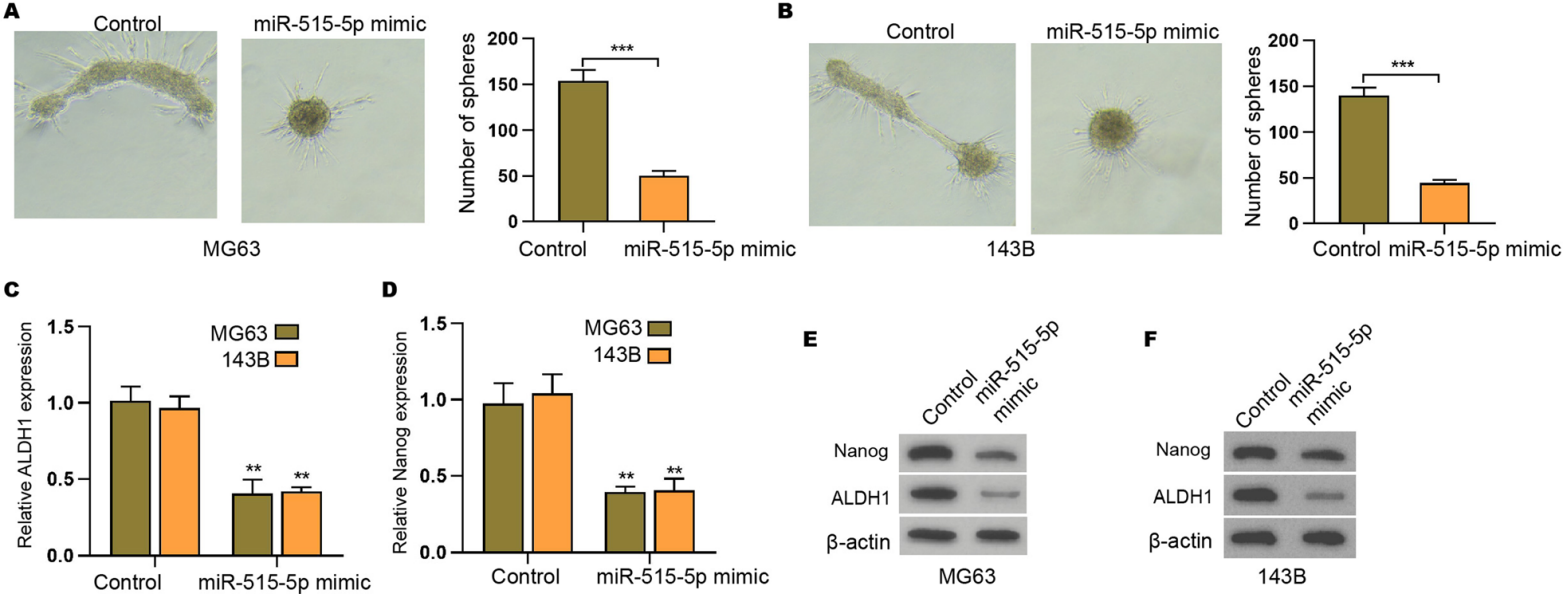
MiR-515-5p inhibits stemness of osteosarcoma cells. **A**–**E** MG63 and 143B cells were transfected with miR-515-5p mimic. **A**, **B** The stemness was analyzed by sphere formation assays. **C**, **D** The mRNA expression of ALDH1 and Nanog was determined by qPCR. **E**, **F** The protein levels of ALDH1 and Nanog were detected by Western blot. Mean  ±  SD (**P  < 0.01, ***P  < 0.001)

Next, we confirmed that the treatment of miR-515-5p mimic attenuated the sphere formation abilities of MG63 and 143B cells (Fig. 5A, B). Meanwhile, the mRNA expression of ALDH1 and Nanog was reduced by miR-515-5p in MG63 and 143B cells (Fig. 5C, D). Moreover, miR-515-5p mimic suppressed ALDH1 and Nanog protein levels in MG63 and 143B cells (Fig. 5E, F).

### YAP is a target of miR-515-5p in osteosarcoma cells

**Fig. 6:**
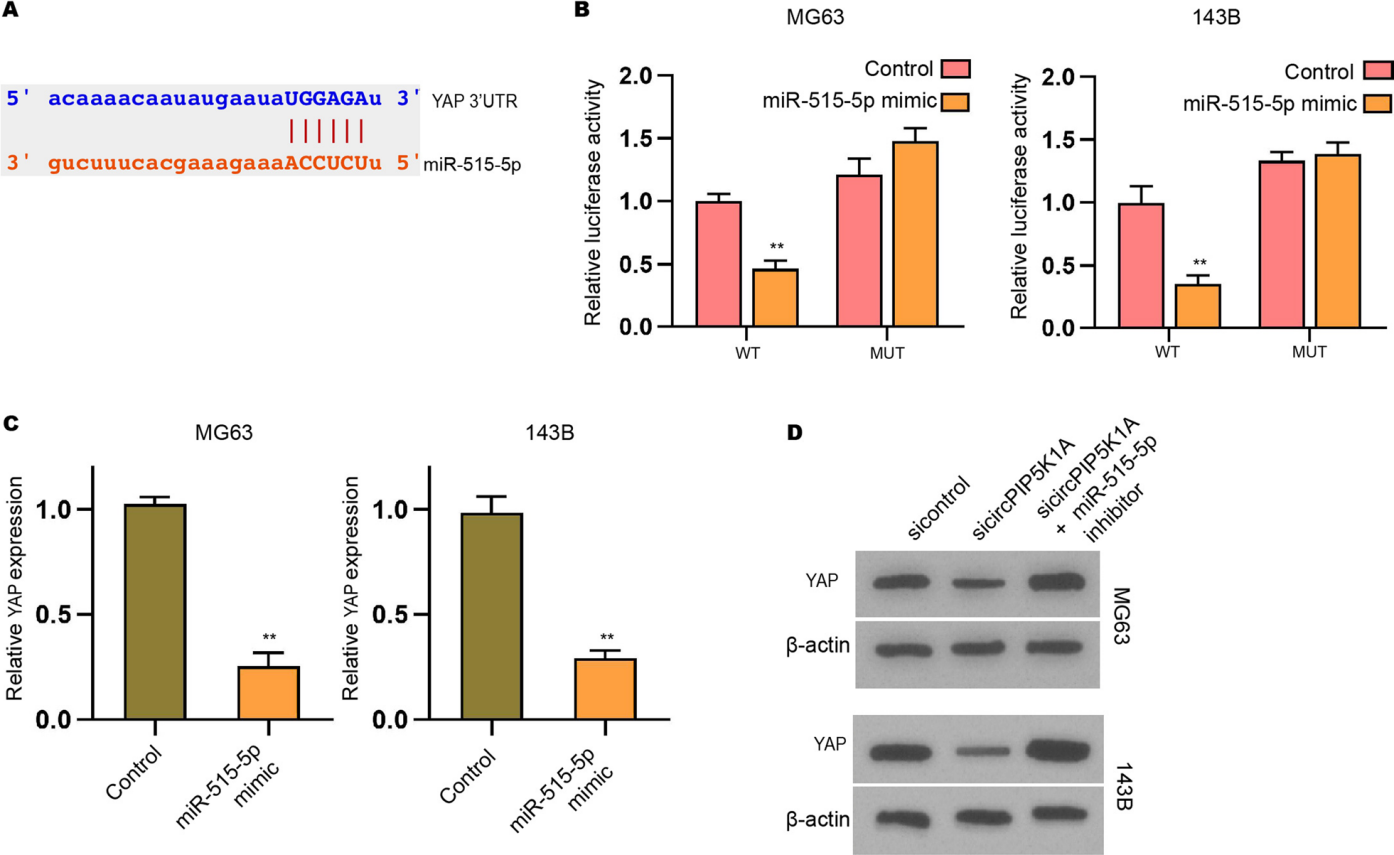
YAP is a target of miR-515-5p in osteosarcoma cells. **A** The interacted binding site between YAP and miR-515-5p. **B**, **C** MG63 and 143B cells were transfected with miR-515-5p mimic. **B** Luciferase activities of YAP or YAP mutant (miR-515-5p binding site) were analyzed by luciferase reporter gene assay. **C** YAP mRNA expression was detected by qPCR. **D** YAP protein levels were tested by Western blot analysis in MG63 and 143B cells transfected with circPIP5K1A siRNA, or co-transfected with circPIP5K1A siRNA and miR-515-5p inhibitor. Mean  ±  SD (**P  < 0.01)

We then found that YAP is one of the potential targets of miR-515-5p and identified the binding site between miR-515-5p and YAP (Fig. 6A). The luciferase activities of YAP 3′UTR, but not YAP 3′UTR with miR-515-5p binding site mutant, were inhibited by miR-515-5p mimic in MG63 and 143B cells (Fig. 6B). The expression of YAP was significantly repressed by miR-515-5p in MG63 and 143B cells (Fig. 6C). Importantly, the inhibition of circPIP5K1A by siRNA reduced YAP expression, while miR-515-5p inhibitor could reverse this reduction in MG63 and 143B cells (Fig. 6D).

### CircPIP5K1A contributes to cancer cell stemness by targeting miR-515-5p/YAP axis in osteosarcoma cells

**Fig. 7:**
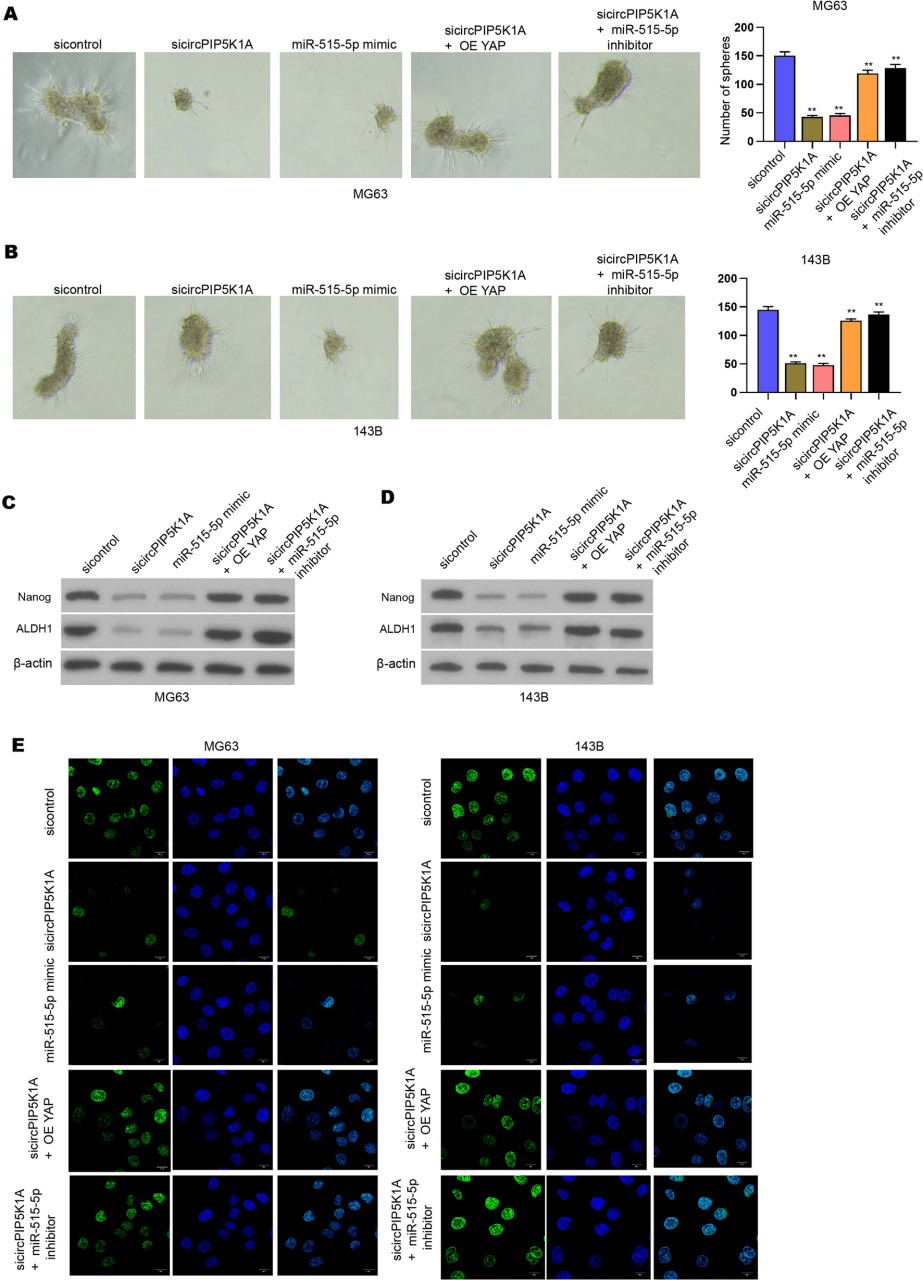
CircPIP5K1A contributes to cancer cell stemness by targeting miR-515-5p/YAP axis in osteosarcoma cells. **A**–**D** MG63 and 143B cells were transfected with circPIP5K1A siRNA or miR-515-5p mimic, or co-transfected with circPIP5K1A siRNA and YAP overexpression vector or miR-515-5p inhibitor. **A**, **B** The stemness was analyzed by sphere formation assays. **C**, **D** The protein levels of ALDH1 and Nanog were detected by Western blot. **E** The expression of Nanog was measured by immunofluorescence analysis. Mean  ±  SD (**P  < 0.01)

Next, we validated the effect of circPIP5K1A/miR-515-5p/YAP axis on the cancer stem cell properties of osteosarcoma cells. We found that the depletion of circPIP5K1A or miR-515-5p mimic could attenuate the sphere formation abilities of MG63 and 143B cells (Fig. 7A, B), and the inhibition of miR-515-5p or YAP overexpression could rescue the attenuation effect of circPIP5K1A MG63 and 143B cells (Fig. 7A, B). Meanwhile, the protein levels of ALDH1 and Nanog were repressed by circPIP5K1A knockdown or miR-515-5p mimic (Fig. 7C–E), while miR-515-5p inhibitor or YAP overexpression reversed circPIP5K1A knockdown-induced repression of the ALDH1 and Nanog expression in MG63 and 143B cells (Fig. 7C, D).

### The depletion of circPIP5K1A represses osteosarcoma cell growth in vivo

**Fig. 8:**
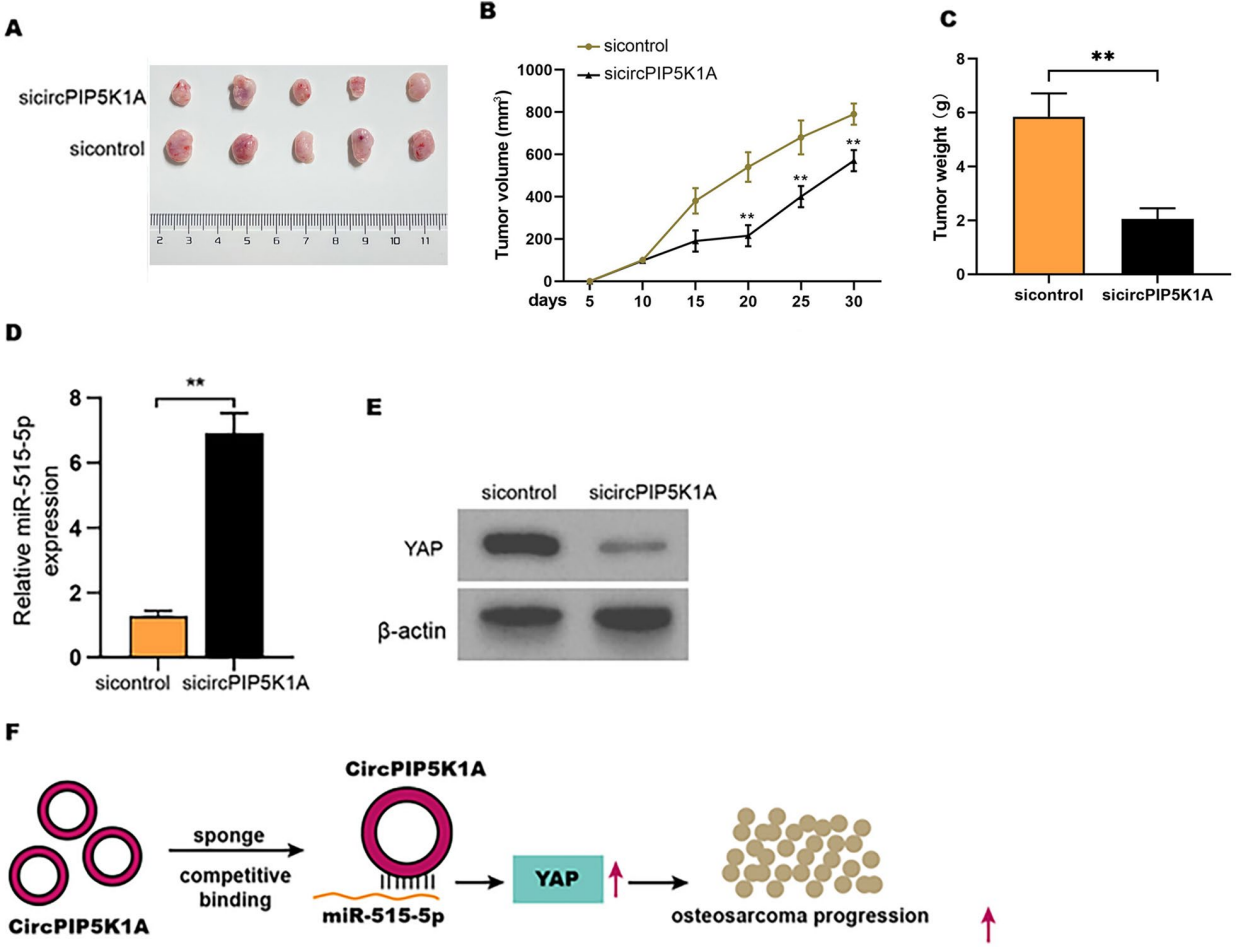
The depletion of circPIP5K1A represses osteosarcoma cell growth in vivo. **A**–**E** Nude mice (n  = 5) were injected with MG63 cells transfected with circPIP5K1A siRNA. The tumor xenograft was conducted in the mice. **A** The tumor images. **B** The tumor volume. **C** The tumor weight. **D** MiR-515-5p expression was measured using qPCR. **E** YAP protein levels were tested by Western blot analysis. **F** A snapshot of this study was shown. Mean  ±  SD (**P  < 0.01)

We then verified the effect of circPIP5K1A on osteosarcoma cell growth in vivo. Tumor xenograft analysis in nude mice demonstrated that the suppression of circPIP5K1A remarkably attenuated the growth ability of MG63 cells in the mice (Fig. 8A–C). The inhibition of circPIP5K1A enhanced miR-515-5p expression in the tumor tissues (Fig. 8D). Besides, the expression of YAP was reduced by circPIP5K1A depletion in the tumor tissues (Fig. 8E).

## Discussion

Osteosarcoma serves as a prevalent kind of bone cancers in children and adolescents. CSCs are self-renewal cancer cells and affect the spread, recurrence, metastasis, and even therapeutic resistance. However, the regulation mechanisms of CSCs in osteosarcoma are still unclear. CircRNAs widely participate in the modulation of cancer progression. In this study, we identified the novel role of circular RNA circPIP5K1A in regulating stemness of osteosarcoma cells.

CircRNAs have been identified to participate in osteosarcoma development and CSCs. Circular RNA circSAMD4A contributes to osteosarcoma cell proliferation via targeting miR-1244/MDM2 expression [20]. Circular RNA circTADA2A enhances metastasis and progression of osteosarcoma by targeting miR-203a-3p to enhance CREB3 [21]. Circular RNA circCPA4/let-7/PD-L1 signaling modulates immune evasion, drug resistance, stemness, and cell growth of in non-small cell lung cancer [22]. Circular RNAs modulate CSCs of hepatocellular carcinoma by competing binding with FMRP against CCAR1 [23]. It has been reported that circPIP5K1A contributes to progression of gastric cancer by miR-376c-3p/ZNF146 signaling [24]. CircPIP5K1A promotes development of ovarian cancer by regulating miR-661/IGFBP5 axis [8]. CircPIP5K1A enhances metastasis and proliferation of non-small cell lung cancer by the regulation of miR-600/HIF-1α [9]. Our data showed that CircPIP5K1A expression was significantly enhanced in clinical osteosarcoma tissues compared with the adjacent normal tissues. The depletion of circPIP5K1A by siRNA repressed osteosarcoma cell viabilities and induced osteosarcoma cell apoptosis. The suppression of circPIP5K1A attenuated the capabilities of invasion and migration of osteosarcoma cells. The circPIP5K1A knockdown increased E-Cadherin expression and decreased Vimentin expression in osteosarcoma cells. The cancer stem cell abilities of osteosarcoma cells were repressed by the depletion of circPIP5K1A. Tumor xenograft analysis in nude mice demonstrated that the depletion of circPIP5K1A represses osteosarcoma cell growth in vivo. These data indicate a new function of circPIP5K1A in the modulation of stemness in osteosarcoma development and present a new evidence of the fundamental roles of circRNAs in osteosarcoma. Due to the close association of CSCs with metastasis and therapeutic resistance, the effect of circPIP5K1A on metastasis and therapeutic resistance in osteosarcoma deserve to investigate in future.

Furthermore, the correlation of YAP with CSCs and osteosarcoma has been identified. The inhibition of HuR represses osteosarcoma cells stemness, invasion, and migration by repressing YAP activation and enhances chemotherapy resistance [25]. Long non-coding RNA B4GALT1-AS1 contributes to stemness and migration of osteosarcoma cells by inducing YAP [26]. Sox2 maintains stemness of cancer cells by regulating the Hippo pathway [27]. It has been reported that miR-515-5p serves as an inhibitor in prostate cancer by regulating TRIP13 [14]. LINC00673 enhances breast cancer cell proliferation by miR-515-5p/MARK4 axis [28]. Circ_0057553/miR-515-5p signaling modulates glycolysis, invasion, migration, apoptosis, and proliferation of prostate cancer cells by regulating YES1 [29]. MiR-515-5p modulates migration of cancer cells by targeting MARK4 [15]. In this study, our data revealed that circPIP5K1A enhanced YAP expression by targeting miR-515-5p. MiR-515-5p inhibited stemness of osteosarcoma cells. The CSCs properties of osteosarcoma cells were repressed by circPIP5K1A knockdown or miR-515-5p mimic, while miR-515-5p inhibitor or YAP overexpression reversed circPIP5K1A knockdown-induced repression. These data present a novel insight into the mechanism involving miR-515-5p/YAP signaling of circPIP5K1A-mediated stemness of osteosarcoma cells. MiR-515-5p is one of the downstream miRNAs of circPIP5K1A and YAP is one of the targets of miR-515-5p. Hence, miR-515-5p/YAP axis may just one of the mechanisms of circPIP5K1A-regulated cancer development. Meanwhile, the clinical relationship of circPIP5K1A, miR-515-5p, and YAP needs to explore in future studies. In addition, we analyzed the stemness markers by Western blot analysis and we will validate it by immunofluorescence in future investigations. Unfortunately, despite the cancer inhibitory function of miR-515-5p has been identified in this investigation and some previous studies, the significance and knowledge of the therapeutic applications of miR-515-5p in metastatic cancers have not been explored, which should be investigated in the future.

## Conclusion

In conclusion, we identified that circular RNA circPIP5K1A contributed to cancer stemness of osteosarcoma by miR-515-5p/YAP axis (Fig. 8F). Targeting circPIP5K1A may be considered as a potential therapeutic strategy for osteosarcoma treatment.

## Data Availability

The datasets used and analysed during the current study are available from the corresponding author on reasonable request.

## Abbreviations

CSCs: Cancer stem cells
circRNA: Circular RNA
miRNAs: MicroRNAs
NSCLC: Non-small cell lung cancer
YAP1: Yes-associated protein1
